# Do macrophages follow the beat of circadian rhythm in TIME (Tumor Immune Microenvironment)?

**DOI:** 10.12688/f1000research.129863.1

**Published:** 2023-01-27

**Authors:** Amelia M. Knudsen-Clark, Juliana Cazarin, Brian J. Altman

**Affiliations:** 1 Department of Microbiology and Immunology, University of Rochester School of Medicine and Dentistry, Rochester, NY, 14620, USA; 2 Department of Biomedical Genetics, University of Rochester School of Medicine and Dentistry, Rochester, NY, 14620, USA; 3 Wilmot Cancer Institute, University of Rochester Medical Center, Rochester, NY, 14620, USA

**Keywords:** Circadian, immunity, tumor immunology, cancer

## Abstract

Advances in cancer research have made clear the critical role of the immune response in clearing tumors. This breakthrough in scientific understanding was heralded by the success of immune checkpoint blockade (ICB) therapies such as anti-programmed cell death protein 1 (PD-1)/ programmed death-ligand 1 (PD-L1) and anti-cytotoxic T-lymphocyte-associated protein 4 (CTLA-4), as well as the success of chimeric antigen receptor (CAR) T cells in treating liquid tumors. Thus, much effort has been made to further understand the role of the immune response in tumor progression, and how we may target it to treat cancer. Macrophages are a component of the tumor immune microenvironment (TIME) that can promote tumor growth both indirectly, by suppressing T cell responses necessary for tumor killing, as well as directly, through deposition of extracellular matrix and promotion of angiogenesis. Thus, understanding regulation of macrophages within the tumor microenvironment (TME) is key to targeting them for immunotherapy. However, circadian rhythms (24-hour cycles) are a fundamental aspect of macrophage biology that have yet to be investigated for their role in macrophage-mediated suppression of the anti-tumor immune response Circadian rhythms regulate macrophage-mediated immune responses through time-of-day-dependent regulation of macrophage function. A better understanding of the circadian biology of macrophages in the context of the TME may allow us to exploit synergy between existing and upcoming treatments and circadian regulation of immunity.

## Introduction

In this review, we discuss circadian regulation of macrophages, how this may contribute to their function in the tumor, and how we can leverage this in the clinic. We will investigate the current literature linking circadian rhythms to the anti-tumor immune response, focusing on macrophages, whose circadian regulation and role in tumor progression have been well-characterized. We will detail how circadian regulation of macrophages may contribute to the anti-tumor immune response, and how the tumor microenvironment (TME) may impact circadian regulation of tumor-associated macrophages. Finally, we will address how circadian immunity may be leveraged to increase efficacy of currently available treatments in the clinic, highlighting recent studies implementing this approach. ^1^^,^^2^ As we are examining the intersection and application of these two fields, we do not provide a comprehensive review of either; both fields have been thoroughly and elegantly reviewed elsewhere. ^3^^–^^5^

## Dysregulation of the immune response occurs in cancer, allowing tumor growth

Tumorigenesis originates with cancer cell-intrinsic factors, yet inflammation and a dysregulated immune response lie at the heart of almost all cancers. ^6^^,^^7^ Tumor growth is associated with the release of immunogenic damage-associated molecular patterns (DAMPs), due to cell stress as a result of mutations and DNA damage, which cause rapid growth and division of cancer cells. In addition, many of the oncogenic mutations acquired by cancer cells promote inflammation through cytokine secretion, which leads to an immune response being directed at the site. ^7^ Through immunosurveillance (the process by which agents recognized as non-self, such as pathogens and pre-cancerous cells, are detected and eliminated), the immune response is critical for the prevention of cancer and tumor clearance, ^8^^–^^12^ and proper coordination can result in elimination of cancer cells. ^13^ However, dysregulation of one or more of the steps in orchestrating the anti-tumor immune response can lead to chronically inflamed areas with “damaged” cancer cells, setting the stage for immune escape. ^3^ Both tumor growth and immune suppression are supported by this low-grade chronic inflammation in the TME, a characteristic that has led to tumors being described as “wounds that do not heal”. ^14^ The chronically inflamed TME is further perpetuated by several different cells within the tumor including macrophages and other myeloid cells, fibroblasts, and the cancer cells themselves, whose secretion of pro-inflammatory cytokines promotes recruitment of different varieties of immune cells, termed leukocytes, to the tumor site. ^15^^–^^17^ Upon recruitment to the tumor, leukocytes are exposed to factors in the TME that inhibit cytotoxic anti-tumor activity, even in the presence of immune stimulatory agents such as DAMPs. ^18^^–^^20^ These factors drive leukocytes to adopt an immunosuppressive phenotype (in the case of macrophages, a pro-resolution state, sometimes referred to as ‘M2’, as opposed to a pro-inflammatory state, sometimes referred to as ‘M1’), which function to promote immune suppression by inhibiting the pro-inflammatory activity of cytotoxic CD8+ T cells, CD4+ T helper 1 cells, and natural killer (NK) cells necessary for tumor cell killing. ^18^^,^^21^ As such, chronic inflammation leads to the persistent activation of inflammatory activity, while paradoxically suppressing the cytotoxic functions necessary for immune-mediated tumor cell killing. ^3^^,^^22^

Immune suppression promotes tolerance, thus becoming unresponsive to an antigen that would otherwise provoke an immune response. This allows cancer cells to escape immunosurveillance. At the same time, macrophages polarized toward a pro-resolution phenotype within the TME can directly promote tumor growth through the secretion of growth factors and proteins that remodel the extracellular matrix. In this way, dysregulation of the anti-tumor immune response by the chronically inflamed TME facilitates immune escape and tumor growth. There has been much success in cancer therapies targeting immune cell function in the tumor by using immune checkpoint blockade (ICB) therapy. One such example are treatments targeted against the inhibitory receptors programmed cell death protein 1 (PD-1) and programmed death-ligand 1 (PD-L1). Chimeric antigen receptor (CAR) T cells also use this approach, with the goal of stimulating the immune response to eliminate the cancer cells. ^23^ However, the degree to which immunosuppressive immune cells infiltrate a tumor and suppress or exclude cytotoxic CD8+ T cells exists in a wide spectrum across cancer. ^23^^–^^25^ This serves to explain why ICB and CAR T cell therapy are ineffective against many solid tumors, and also why targeting immunosuppressive myeloid cells such as macrophages in combination with ICB has become of interest.

## Circadian control of macrophage function contributes to regulation of immune responses

Circadian rhythms are 24-hour rhythms that drive oscillations in the levels of circadian-regulated gene transcripts and proteins in a tissue- and cell-specific manner. ^26^^,^^27^ This results in time-of-day-dependent variations in circadian-regulated cell processes and functions ^28^ that are maintained by the molecular clock, which is formed by a series of transcription/translation feedback loops. ^28^ Molecular clocks are present in almost every cell of the human body and are synchronized by signals sent out from the central clock, which entrains molecular clocks to the time of day. ^28^ This results in the temporal coordination of cells in spatially distinct tissues, and thus tissue functions. ^28^ While peripheral clocks receive entraining signals from the central clock, they do not rely on these external signals to maintain rhythmicity and will continue to oscillate in the absence of external cues. In mouse experiments, the time of the day with respect to circadian rhythm is usually marked by ‘Zeitgeber time’ (ZT), which corresponds to when the lights are turned on and off. ZT0 corresponds to when the lights are turned on (usually 7 AM) and ZT 12 corresponds to when the lights are turned off (usually 7 PM). It is important to note that since mice are nocturnal, daytime / lights on corresponds to their inactive phase when they sleep, and nighttime/lights off corresponds to their active phase when they are most active and eat the most. This is opposite in humans, as we are diurnal. For the purposes of this review, we will use the terms “active phase” and “inactive phase” to refer to circadian experiments.

It has been suggested that circadian regulation of immune responses has evolved in part to leverage the benefit of inflammatory responses ( *i.e.*, elimination of pathogens) while reducing the costs incurred from the drawbacks of inflammation ( *i.e.*, tissue damage). ^4^ Current data indicate that macrophage-mediated immune responses are elevated at the end of the inactive phase, potentially to prime responses during the time when an organism is active (in humans, corresponding to daytime), allowing heightened protection against environmental insults and pathogens when encounter is most likely. ^5^^,^^29^^,^^30^ Conversely, macrophage-mediated immune responses are dampened during the time when an organism is at rest (in humans, corresponding to nighttime), and encounter with environmental insults and pathogens is least likely. ^31^^,^^32^ In this way, immune responses are tuned, but not restricted, to different times of day. However, this is a general framework for some infection models that does not apply to all cases. Notably, regulation of inflammation ( *i.e.*, interferon responses) and immune cell recruitment is also regulated by non-immune structural cells in distinct organs, with evidence that this may manifest in tissue-specific differences in timing of peak inflammatory response. ^33^^,^^34^ Conceivably, this could reflect a mechanism through which circadian regulation optimizes tissue-specific responses, as different parts of the body are susceptible to different pathogens/insults at different times of day.

Such modulation of the magnitude of inflammatory response is key, as tissue damage from inflammation can be pathogenic. Tissue damage in the absence of pathogen is considered sterile inflammation and is important for tissue repair. ^35^^,^^36^ However, if sterile inflammation is not well-managed and resolved in a timely manner, it results in chronic inflammation, increasing risk of immune dysregulation that can ultimately lead to autoimmunity or cancer. ^6^^,^^35^^,^^37^ Thus, inflammatory activity must be self-limiting, so as to reach a natural resolution of inflammation and return to homeostasis. At the same time, the immune response must be strong enough to clear the pathogen so that it can be cleared quickly. Otherwise, elongation of the duration of inflammatory response can also promote immune regulatory mechanisms to minimize tissue damage. This is particularly relevant for cancer, as the lack of a strong enough response can contribute to failed immunosurveillance. Failed immunosurveillance can lead to cancer formation and progression. Given the emerging yet evident role of circadian regulation in coordination of immune responses, better understanding this additional layer of regulation on immune cell function in health and disease will better inform on how best to target for treatment.

**Figure 1.**
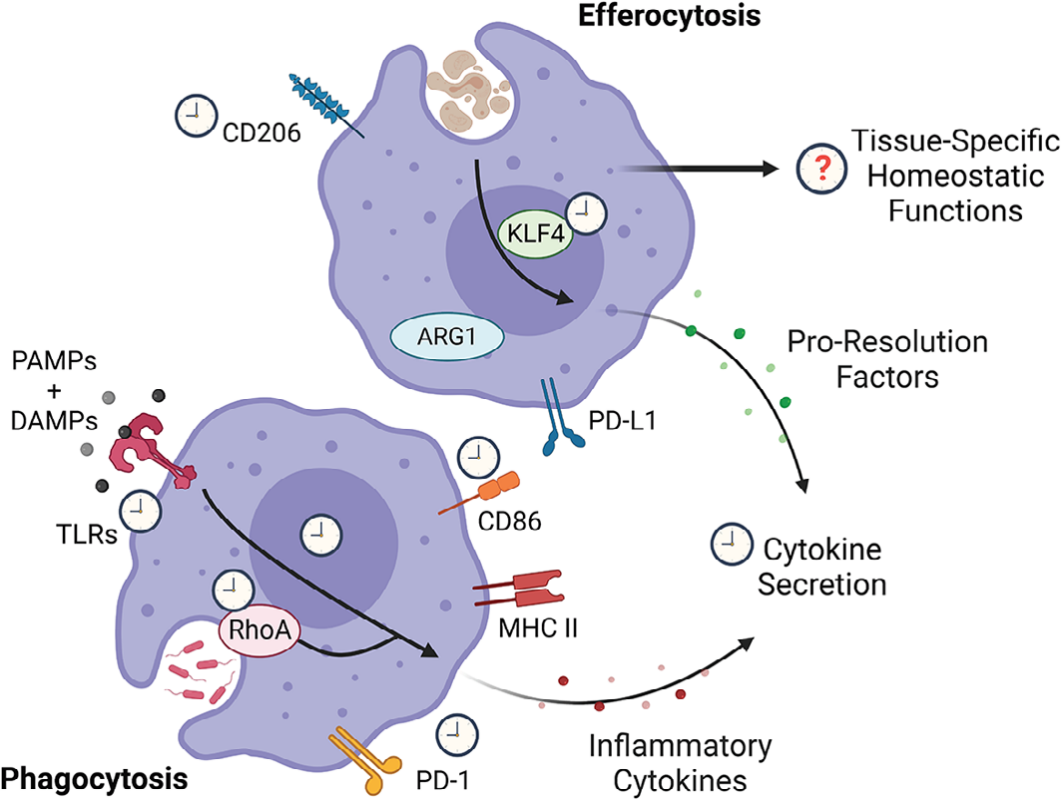
Key aspects of macrophage function are under circadian regulation. While the potential circadian regulation of various aspects of macrophage biology remain unknown, proteins downstream of pathogen sensing confer a circadian signature on the magnitude of cytokine secretion. KLF4, which promotes silent clearance of cells *via* a pro-resolution profile on macrophage following efferocytosis has also been found to be circadian. RhoA, which is key to phagocytosis, appears to be circadian-regulated, but whether phagocytosis is circadian-regulated remains unclear. Figure created with BioRender.com. KLF4, Krüppel-like factor 4; RhoA, Ras homolog family member A; PD-L1, programmed death-ligand 1; ARG1, Arginase 1; PAMP, pathogen-associated molecular patterns; DAMP, damage-associated molecular patterns; TLR, toll-like receptors; PD-1, programmed cell death protein 1; MHC II, major histocompatibility complex class II.

Circadian rhythms regulate key aspects of macrophage function ( Figure 1), but the extent to which this is relevant in cancer is unknown. As we will discuss below, there is some evidence that circadian regulation of macrophages is disrupted in tumor-bearing mice. However, more studies are required to determine whether circadian regulation of immunity is intact systemically or within the tumor, and if it remains entrained to the day/light cycle across different types of cancer and in humans. Understanding how circadian rhythms influence macrophage-mediated immune suppression may inform on new approaches to cancer treatment.

In this review, we highlight the role of macrophages in tumor biology and the anti-tumor immune response. In order to understand how the immune response is dysregulated in the TME, we must first understand the role of macrophages during homeostasis and in response to inflammation, and how circadian regulation contributes to this. We then discuss the role of macrophages in tumor development and the anti-tumor immune response, shedding light onto how they are dysregulated in the TME. Finally, we review the current literature on circadian regulation of macrophages and discuss how this may contribute to their function in the tumor.

## Macrophages are circadian-regulated innate immune cells with diverse functions that are major contributors to immune suppression within the TME

One of the most abundant leukocytes found in many solid tumors are tumor-associated macrophages (TAMs). ^38^ Macrophages are tissue-resident immune cells (leukocytes) of the myeloid lineage that are present in all organs. As professional phagocytes, macrophages are specialized in their ability to phagocytose dead and dying cells in the body through a process called efferocytosis (discussed in “Efferocytosis”, below). Macrophages respond to diverse environmental signals to adopt tissue-specific phenotypes; as such, macrophages are shaped by their environment, enabling them to perform a wide variety of tissue-specific homeostatic functions. ^39^^,^^40^ Due to their ability to sense and respond to the environment, macrophages play a key role in coordinating the immune response, responding rapidly to infection or tissue damage.

### Cytokine secretion

Time-of-day-dependent macrophage response to stimuli, conferred by the molecular clock, can modulate the magnitude of the resulting adaptive immune response and determine disease progression. ^31^^,^^41^^,^^42^ Upon sensing pathogen-associated molecular patterns (PAMPs) and DAMPs, macrophages can become activated, adopting a pro-inflammatory phenotype. Pro-inflammatory macrophages are broadly characterized by upregulation of phenotypic markers major histocompatibility complex class II (MHC II) and CD86 on the cell surface, and secretion of pro-inflammatory cytokines such as IL-6, IL-1β, TNFα, and IL-12. However, this response is tuned by a variety of factors including environment, pathogen type, and even pathogen strain; thus, *in vitro*-characterized markers of macrophage phenotype and the M1/M2 paradigm are only to be viewed as a loose guideline. ^43^^,^^44^ Upon activation, macrophages secrete pro-inflammatory mediators to initiate an immune response, recruiting circulating leukocytes (neutrophils, monocytes, NK cells) to the site of infection. In the context of acute inflammation, this can help eliminate pathogen. However, in a chronically inflamed environment such as in cancer, recruited immune cells such as macrophages can be driven to instead perform immunosuppressive functions. Thus, continued recruitment of these cells to the chronically inflamed TME is one way in which macrophages can contribute to immune suppression.

Chemokine and cytokine secretion by macrophages in response to stimuli is subject to circadian regulation. ^31^^,^^42^^,^^45^^–^^47^ Peak macrophage response to stimuli varies, likely due to tissue-specific and pathogen-specific context. ^34^^,^^45^^,^^47^^–^^49^ Collectively, current data indicate that stimulation of macrophages between the mid-inactive phase and the early active phase (ZT6-15) induces increased secretion of pro-inflammatory cytokines and chemokines compared to when stimulation occurs between the late active and early inactive phase (ZT18-3). ^31^^,^^42^^,^^45^^–^^47^^,^^49^ By contrast, secretion of the anti-inflammatory cytokine IL-10 has been observed to be higher when stimulated during the early inactive phase (ZT0) than the early active phase (ZT12). ^46^ Data indicate this is driven by a molecular predisposition toward pro-inflammatory activity at a certain time of day, ^42^^,^^46^^,^^50^^,^^51^ with some evidence that macrophages may indeed be poised for activation at a certain time of day. ^42^ Additionally, well-known neuroendocrine timing signals controlled by the central clock, including the adrenergic system and oscillating levels of serum glucocorticoids and melatonin have been shown to modulate macrophage function or differentiation, suggesting that multiple signals are potentially contributing to macrophage circadian control. ^52^^–^^55^ Whether this is simply a mechanism to limit macrophage-mediated inflammatory activity during late active/early inactive phase, or whether this translates more broadly to circadian patterns in macrophage phenotype and function at homeostasis requires further study. Importantly, future work characterizing which pathways in macrophages are under circadian control must focus on changes at the protein and phospho-protein level, as circadian patterns in mRNA transcripts do not always equate to circadian patterns in levels of the encoded protein. ^56^

Of note, the majority of studies interrogating circadian control of macrophage function thus far have been performed using lipopolysaccharide (LPS) exposure or bacterial infection as a model of acute inflammation. More studies are required to determine whether these findings apply across the full spectrum of pathogens, although the limited studies using non-bacterial models of acute inflammation are in line with these findings. ^49^^,^^57^ There is some evidence that this time-of-day macrophage response extends to stimulation by DAMPs. ^34^^,^^41^ The response of macrophage to stimuli is highly context-dependent based on receptor signaling; while in part determined by expression levels of the receptor, it is also dependent on downstream signaling. ^58^ Indeed, few pattern recognition receptors (PRRs) have been observed to be circadian in macrophages; instead, data indicate that circadian patterns in macrophage response to stimuli are broadly conferred by circadian regulation of proteins involved in signaling downstream of PRRs. Thus, circadian regulation of proteins involved in signaling downstream of PRRs as well as of the PRRs themselves could represent a way to regulate by time of day not only ability to sense pathogen, but the activation threshold for PRR signaling.

### Phagocytosis

Aside from recruiting cells to the site of infection, macrophages can promote clearance of extracellular pathogens through phagocytosis. There is ample evidence for circadian regulation of phagocytosis, but a wide range of findings in the literature make this a subject of continued debate. ^42^^,^^47^^,^^59^^–^^62^ The clearest evidence of the mechanism driving circadian regulation of phagocytosis in macrophages is through regulation of Ras homolog family member A (RhoA) activity, which promotes phagocytosis, downstream of the molecular clock. ^42^ However, an extensive study performed by a separate group showed evidence that there were no *in vivo* rhythms in phagocytosis and that, furthermore, rhythms in phagocytosis observed *ex vivo* were independent of the macrophage molecular clock. ^47^ As a result, the role of circadian rhythms in phagocytic activity of macrophages in response to stimuli remains unclear.

Direct clearance of pathogens by macrophages is not limited to extracellular pathogens; macrophages can promote clearance of intracellular pathogens by efferocytosis of infected cells. ^63^ In doing so, the pathogen within the infected cell is contained and degraded, and can stimulate PRRs including toll-like receptors (TLRs) in macrophages to trigger a pathogen-specific response.

### Efferocytosis

A key homeostatic function of macrophages, efferocytosis is a highly coordinated process involving the detection of dead and dying cells by “find me” signals, engulfment through “eat me” signals, processing, and context-dependent response. ^64^ This is distinct from phagocytosis of pathogens. If not cleared from tissue in a timely manner, apoptotic cells can undergo secondary necrosis and become highly inflammatory. ^65^ Under homeostatic conditions, efferocytosis can trigger macrophages to release factors that suppress inflammation and promote tissue repair, maintaining homeostasis during normal tissue turnover. ^66^ As a result, macrophages are uniquely well-equipped to protect tissue integrity under normal homeostatic conditions. ^66^^,^^67^ However, as we will discuss (below in “Macrophages in tumorigenesis”), this process can be taken advantage of by cancer cells to evade immunity.

Importantly, the pathways involved in efferocytosis are distinct from those utilized when phagocytosing extracellular pathogens. ^62^ RhoA activity can preferentially facilitate phagocytosis by suppressing efferocytosis. ^62^^,^^68^ Thus, regulation of RhoA by the molecular clock could conceivably present a mechanism through which a predisposition toward phagocytosis (of extracellular pathogens) or efferocytosis (of cells) could be imposed on macrophages at distinct times of day. Nonetheless, efferocytosis is a highly coordinated process that involves several steps prior to engulfment, such as recognition of “find me” and “eat me” signals ^69^; phagocytosis as well occurs downstream of “tasting” and “feeling” the target. ^70^ Better understanding whether receptors and signaling mediators involved in efferocytosis and phagocytosis are circadian-regulated will help elucidate how circadian regulation may contribute to this key aspect of macrophage function during health and disease.

Efferocytosis is of particular importance to clear neutrophils, which must be cleared daily due to their short lifespan. Circadian variations in efferocytosis under homeostatic conditions have been observed, with neutrophil-engulfing macrophages more frequent in peripheral tissues at the end of the inactive phase in mice (ZT11) than at the beginning of the inactive phase (ZT3). ^60^ Whether these circadian rhythms in efferocytosis at homeostasis are regulated directly through the macrophage molecular clock, or indirectly as a result of circadian patterns in neutrophil trafficking is unclear and requires further study. Of note, expression of genes encoding proteins involved in efferocytosis ^71^ followed a similar circadian pattern in peritoneal macrophages, as did the frequency of CD206+ splenic macrophages under homeostatic conditions – higher at the end than the beginning of the inactive phase. ^50^^,^^72^ Further studies are required to determine the significance of circadian regulation of efferocytotic machinery with respect to coordination of macrophage function at different times of day. ^73^

Tissue environment imprints macrophage function and phenotype, and even within a given tissue, different populations of macrophages can be specialized toward certain functions. ^58^^,^^73^^,^^74^ As a particularly relevant example, a subset of macrophages within certain tissues were found to be pre-programmed by the tissue microenvironment to silently clear apoptotic cells, which is important for suppressing inflammation during homeostasis. ^58^ This non-inflammatory clearance of apoptotic cells was promoted by decreased expression of the Toll-like receptor TLR9, which has been shown to be circadian-regulated, ^31^ coupled with increased expression of negative regulators of TLR signaling. Expression of receptors recognizing apoptotic cells was also increased in these macrophages; as a result, apoptotic cells were predominately engulfed by macrophages that were pre-programmed to be less sensitive to TLR agonists associated with apoptotic cells such as DNA and RNA, decreasing the likelihood of an inflammatory response. This was driven in part by expression of Krüppel-like factor 4 (KLF4) and KLF2, which control expression of genes important for silent clearance of apoptotic cells. KLF4 was found to be diurnally expressed in peritoneal macrophages at homeostasis, peaking at the end of the inactive phase (ZT12). ^61^ The potential contribution of circadian expression of KLF4 to maintenance of homeostasis by non-inflammatory clearance of apoptotic cells is unknown. Whether KLF4 expression is circadian-regulated in the whole population of peritoneal macrophages or if the observed circadian variation in expression was driven by a subset of macrophages within the peritoneal cavity remains to be seen, as does whether KLF4 expression is circadian in macrophages of other tissues. It is not illogical that certain functions or responses to certain pathogens may be circadian regulated in macrophages of certain tissue but not in others, as certain tissues, such as barrier tissues, may be more at risk of being exposed to certain pathogens. This is supported by the observation that expression of certain PRRs have been found to be circadian in some macrophage populations but not in others. Certainly, in order to gain a broader understanding of how circadian regulation of macrophages contributes to homeostasis and host defense, it will be important to characterize macrophages from diverse tissues, with attention to subpopulations within the tissue itself.

The risk of cancer and other chronic inflammatory diseases is higher in older individuals. Aging was recently found to diminish circadian rhythms in macrophages, in a manner that might negatively affect rhythmic efferocytosis. In macrophages of aged mice, diurnal patterns in KLF4 expression were muted, leading to decreased KLF4 expression at times of day it would otherwise be heightened. ^61^ This was coupled with a general loss of circadian transcriptional control and rhythmic phagocytosis in macrophages from aged mice. Of note, it has been shown that macrophages from older individuals tend to be more inflammatory at baseline, promoting chronic inflammatory conditions, yet less capable of elevating inflammatory activity to mediate bacterial clearance, leading to susceptibility to infection in older populations. Further studies are required to determine if loss of circadian rhythmicity and an increased propensity towards inflammation in aged macrophages could contribute to tumorigenesis.

In the context of cancer, efferocytosis of cancer cells is one way in which macrophages can obtain tumor antigen to present to intra-tumoral T cells. However, in the immunosuppressive TME, an immunosuppressive or pro-resolution phenotype is often promoted such that presentation of antigen can suppress cytotoxic responses and confer tolerance. Whether the potential circadian regulation of macrophage efferocytosis would apply in the context of inflammation is unknown and may be something to consider when assessing the contribution of macrophages to rhythms in clearance of intracellular pathogens.

Aside from effector functions to clear pathogens, macrophages can also present antigen to T cells at the site of inflammation. This promotes optimal T cell effector functions. Data indicates that expression of CD86, a co-stimulatory protein involved in T cell activation, is circadian-regulated in macrophages. ^50^^,^^72^ Of note, macrophages upregulate CD86 when upon activation, and CD86 has also been used as a phenotypic marker for pro-inflammatory macrophages. This may be yet another layer of circadian regulation on macrophages that prime them for activation at certain times of day.

### Metabolic programming of macrophages

Macrophage phenotype and function is highly dependent on metabolic programming, in that macrophage function is limited in its effectiveness without the required metabolic switch to support these functions. ^75^^,^^76^ To accommodate effector functions that are highly energetic and must be quickly produced (like sprinting instead of running a marathon), upon activation macrophages undergo a metabolic switch to glycolysis. ^77^ By contrast, oxidative phosphorylation is prioritized when macrophages are polarized toward a pro-resolution phenotype. ^78^ Macrophage metabolism is less glycolytic under homeostatic conditions than upon activation, but the main pathways utilized appear to vary by tissue, likely due to tissue-specific functions. ^75^ Circadian gating of macrophage response to stimuli is in part facilitated by circadian modulation of macrophage metabolism. ^56^ Data indicates that circadian modulation of macrophage metabolism confers an increased capacity for glycolytic shift following stimulation at the end of the inactive phase. ^41^^,^^79^^,^^80^ This corresponds with the current data on macrophage response to stimuli, collectively indicating a multi-pathway role for circadian regulation of macrophage response to stimuli through time-of-day-dependent macrophage metabolism and signaling pathways downstream of PRRs. Additionally, nutrient availability is circadian: several studies have shown that metabolites oscillate in circulation in a manner that can be disrupted by shift-work or sleep disturbance, ^81^^–^^83^ but the role of this in gating macrophage polarization or activation has not yet been determined. Whether circadian regulation of metabolism influences macrophage function during homeostasis (for example, balance of efferocytosis *vs.* tissue-specific functions) remains unknown.

In line with metabolic control of macrophage function, metabolism contributes to the various feedback mechanisms that are in place to promote self-limiting inflammation. When there are many active (and thus highly glycolytic) immune cells in a location, such as pro-inflammatory macrophages and activated CD4 and CD8 T cells, the local microenvironment will become more acidic/high in lactate, which feeds back to suppress glycolysis. ^84^ This limits pro-inflammatory effector function and promotes a metabolic switch to oxidative phosphorylation. Coupled with metabolism-mediated negative feedback mechanisms on inflammatory function are immunologic feedback mechanisms. Macrophages can upregulate PD-1 upon activation, and engagement of PD-1 with the ligand PD-L1 (through interaction with other cells) can promote a switch back to oxidative phosphorylation, supporting a pro-resolution phenotype. ^85^^,^^86^ Macrophages can also upregulate PD-L1 upon activation, which can suppress inflammatory activity of other PD-1 expressing-cells. ^41^^,^^87^ Lactate, which increases following activation due to glycolysis, further upregulates PD-L1 expression, acting as a feedback loop to limit inflammatory activity. ^88^ As inflammation continues, tissue damage increases, and with it the presence of apoptotic cells. ^71^ Engulfment of uninfected apoptotic cells induces a pro-resolution phenotype in macrophages. Among other things, this promotes secretion of IL-10, which further feeds back to suppress the glycolytic metabolism needed to support inflammatory functions – thereby creating a feed-forward loop that reinforces the shift to the resolution of inflammation. ^89^ Normally, these feedback mechanisms function to limit the pathogenic effects of the inflammatory immune response and promote the natural resolution of inflammation. However, as we will discuss below, these feedback mechanisms to limit inflammation are co-opted by cancer to create an immunosuppressive TME.

### Contextual cues dictate macrophage function

As pathogen or target mammalian cell is cleared by phagocytosis or efferocytosis, cues in the microenvironment such as the absence of PAMPs in combination with increased lactate, acidity of the environment, and apoptotic cells drive macrophages to downregulate inflammatory functions and adopt pro-resolution functions. ^84^^,^^90^^,^^91^ Pro-resolution macrophages have been characterized by expression of CD206 and Arginase 1 (ARG1), but *in vivo* they exist across a spectrum of phenotypes that are dependent on many factors, including tissue environment and stage of resolution. ^90^ During the resolution phase, macrophages promote tissue repair by secreting growth factors and proteases to remodel the extracellular matrix, such as VEGF and MMPs. Clearance of dead cells by efferocytosis removes potentially inflammatory cell debris from the environment, suppressing further inflammation. Efferocytosis reinforces an immunoregulatory (pro-resolution) phenotype in macrophages by suppressing TLR signaling and inducing ARG1 expression and secretion of anti-inflammatory factors such as IL-10, TGFβ, and PGE2. ^71^^,^^92^^,^^93^ These anti-inflammatory proteins can suppress inflammatory activity of other cells. There is a dearth of studies on the role of circadian regulation in macrophage-mediated resolution of inflammation and homeostatic functions. Given the key role macrophages play in maintaining homeostasis and resolution of inflammation, this merits further investigation. As we will discuss, the TME fosters adoption of these pro-resolution and anti-inflammatory functions of macrophages, which are key drivers of tumor growth and immune suppression in the TME.

Disruption of the molecular clock in myeloid cells predisposes mice to developing exacerbated inflammatory responses in both acute and chronic inflammatory disease models and can lead to impaired resolution of inflammation. ^42^^,^^46^^,^^94^ This suggests that circadian regulation of macrophages by the molecular clock functions to restrict inflammatory activity, thereby promoting resolution of inflammation. This is of particular interest considering that tumor growth and immunosuppression within the TME are both promoted by chronic inflammation. However, how circadian control of macrophages influences immune suppression in the TME remains unknown.

## Macrophages in tumorigenesis

**Figure 2.**
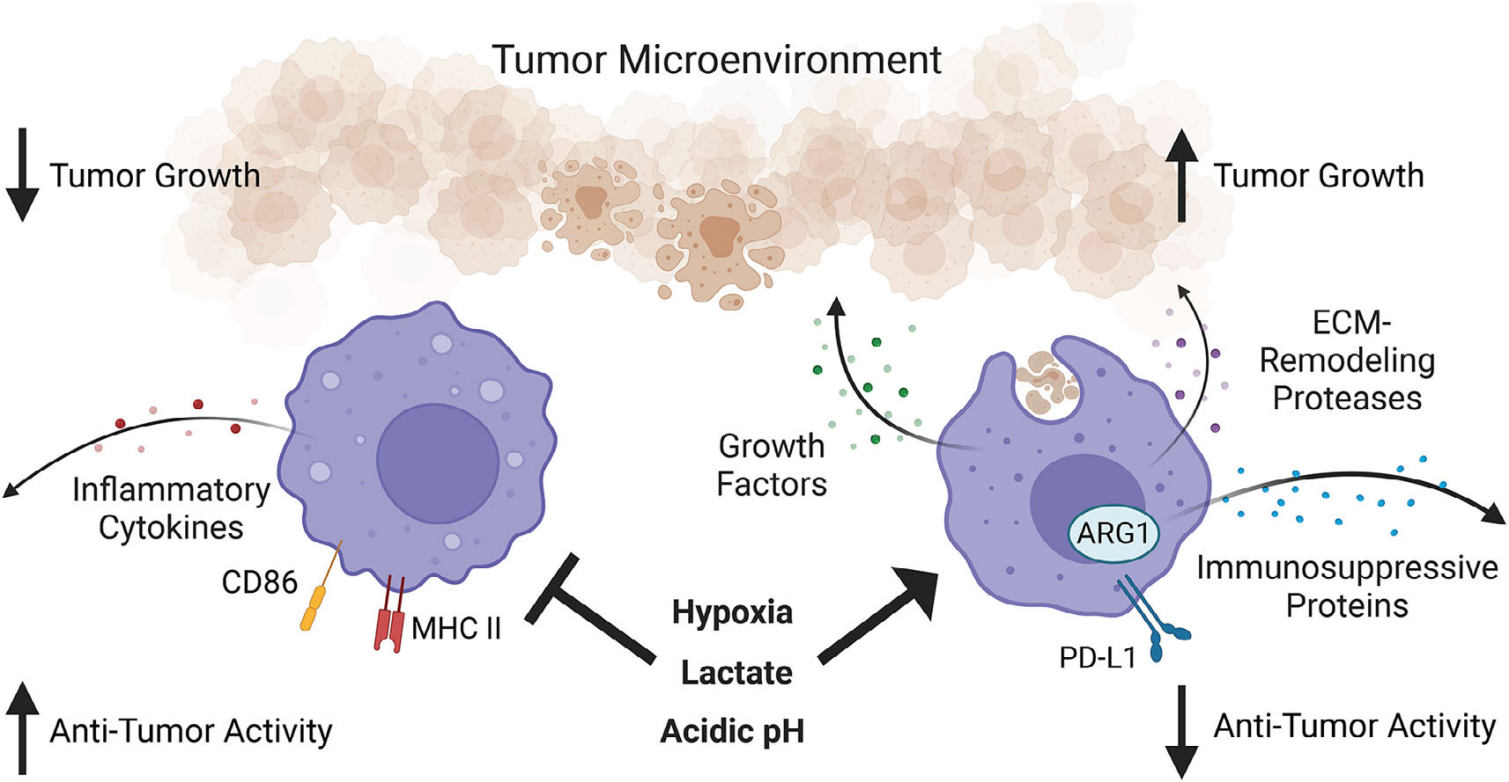
Macrophages in tumorigenesis. While tumor-associated macrophages are heterogeneous, they are overall immunosuppressive due to the tumor microenvironment. Hypoxia, lactate, and acidic pH in the tumor microenvironment promote pro-growth, tissue repair, and immunosuppressive functions of tumor-associated macrophages while suppressing their pro-inflammatory functions. What aspects of macrophage function in the tumor microenvironment remain under circadian regulation is an open question. Figure created with BioRender.com. ARG1, Arginase 1; MHC II, major histocompatibility complex class II; PD-L1, programmed death-ligand 1; ECM, extracellular matrix.

Within the tumor, macrophages can phagocytose tumor cells, present tumor-associated antigen to intratumoral T cells, and secrete pro-inflammatory cytokines such as TNFα and IL-12 to promote T cell activity and effector function ( Figure 2). ^95^ However, macrophage activation and inflammatory activity is limited in the TME. This is largely due to the subversion of immune regulatory mechanisms in the chronically inflamed TME. While immunogenic DAMPs can elicit an inflammatory response from macrophages, chronic stimulation of TAMs by DAMPs can promote an immunoregulatory phenotype. ^18^ Efferocytosis of apoptotic cells in the TME can also induce immunoregulatory functions and suppresses activation of TAMs. Along with chronic stimulation and abundance of apoptotic cells, various metabolic factors in the TME disrupt immune homeostasis to suppress inflammatory activity and promote tissue repair/wound-healing functions to facilitate restoration of homeostasis. ^96^ These factors include hypoxia, acidity, high lactate, and release of adenosine from dying tumor cells. ^20^^,^^97^^–^^99^ These are related to: a) poor vascularization, leading to poor oxygen flow and nutrient delivery, b) chronic inflammatory activity of cells in the TME and rapid growth of cancer cells, all of which is supported by glycolytic activity – leading to excretion of lactic acid, acidifying environment, and potentially depletion of glucose, and c) poor drainage of the TME due to poor vasculature and lymphatics, leading to the accumulation of waste products instead of being washed away/drained. Metabolic heterogeneity of different TAM subsets may also contribute to how they behave in the TME. As an example, a recent study showed that pro-resolution TAMs can utilize lactate as a fuel source. while, in contrast, elevated lactate concentration can decrease the glycolytic activity of pro-inflammatory TAMs. ^100^

Macrophage inflammatory activity is also modulated by factors secreted by cells within the tumor, including infiltrating immune cells, fibroblasts, and the cancer cells themselves. Anti-inflammatory factors such as IL-10 and PGE2 secreted by other immune-infiltrating cells and the tumor cells themselves suppress inflammatory activity. ^101^ Type 2 CD4+ T helper cells can secrete IL4/IL13, which promotes a pro-resolution macrophage phenotype, suppressing a pro-inflammatory phenotype. ^102^ Cancer-associated fibroblasts can deposit collagen, increasing extracellular matrix (ECM) density, which can promote a pro-resolution phenotype in macrophages. The degree to which macrophages are exposed to each of these factors in the TME varies within tumors depending on several factors including the distance from blood vessels and neighboring cells. ^103^^,^^104^ Thus, phenotype is dependent on the location of the macrophage within the TME, due in part to the ability of macrophages to sense and adapt to the local microenvironment. As a result, there is significant phenotypic heterogeneity of TAMs within tumors ^105^^,^^106^; while regions of the TME confer immunosuppressive or pro-tumorigenic functions to TAMs, there are regions in the tumor where anti-tumorigenic TAMs are found as well. ^107^^,^^108^ Traditionally, anti-tumor/pro-inflammatory macrophages have a phenotype associated with expression of MHCII and CD86 and secretion of TNFα. By contrast, pro-tumor/anti-inflammatory TAMs have a phenotype associated with expression of CD206, ARG1, PD-L1 and secretion of IL10. However, in practice the TAMs exist in the tumor across a spectrum of these phenotypes, which, along with their role/function in the tumor, can vary across different types of cancer. ^109^ Despite the heterogeneity of TAMs both across and within tumors, overall high intra-tumoral TAM density generally correlates with poor prognosis/poor patient outcome across cancer types, ^96^^,^^110^^,^^111^ suggesting that the bulk of macrophages within the tumor are immune suppressive/pro-tumorigenic.

TAMs can promote tumorigenesis directly, through supporting tumor growth and metastasis, and indirectly, through suppression of the anti-tumor immune response. In the context of the chronically inflamed TME, failure to resolve the inflammation can lead to uncontrolled secretion of pro-resolution tissue repair factors by TAMs, which promote tumor growth and metastatic capacity. ^37^^,^^112^^,^^113^ Secretion of VEGFa can promote angiogenesis, accommodating tumor growth/increased tumor mass by supplying it with more nutrients. ECM remodeling proteases such as MMP8-10 can promote metastasis by motility and invasiveness of cancer cells. TAM-derived TGFβ can promote activation of cancer-associated fibroblasts and together, through ECM remodeling and increased ECM deposition, they can promote exclusion of CD8+ cytotoxic T cells from the tumor, facilitating immune evasion.

Within the tumor, there can be an abundance of apoptotic cells due to the metabolically stressful TME and pathogenic effects of chronic inflammation. Efferocytosis of apoptotic cells by macrophages induces expression of immunoregulatory proteins IL-10, TGFβ, and ARG1 to dampen inflammation, promoting suppression of CD8+ T cell cytotoxic activity. ^18^^,^^92^^,^^93^^,^^101^^,^^114^ TAMs can also inhibit cytotoxic lymphocyte activity through expression of the checkpoint inhibitor PD-L1, upregulation of which is promoted by hypoxia and lactic acid. ^88^^,^^109^^,^^115^ Expression of PD-L1 by TAMs has been shown to be pro-tumorigenic and a poor prognostic indicator. There is recent evidence that PD-L1 is indirectly regulated by the molecular clock in macrophages downstream of PKM2, which has been shown to promote PD-L1 expression. ^41^^,^^87^ Whether PKM2 expression by TAMs is circadian in tumors, and whether this translates to circadian variations in PD-L1 expression, remains to be seen.

Recent studies have begun to decipher the potential role of the macrophage circadian clock in specifying TAM roles in the TME. In B16-F10 (melanoma) tumor-bearing mice, the time of day variation in CD86+ splenic macrophage frequency was lost, which suggests that systemic cues in tumor-bearing mice can alter circadian rhythms of macrophages in distal peripheral tissues. Of note, in the tumor itself, circadian variations in frequency of CD86+ macrophages was observed, but the peak of CD86+ cells was reversed from in peripheral tissues. ^72^ It was not clear if this circadian variation in CD86 was due to circadian oscillation of phenotype in the TAMs (from a pro-inflammatory to a pro-resolution phenotype) or due to rhythmic recruitment of macrophages that adopted different polarization states. This discordance with the time-of-day peak and trough of macrophages in tissues of healthy mice (spleen, peritoneal cavity) ^50^^,^^72^ may suggest a disruption in circadian regulation of macrophages within the tumor microenvironment, perhaps through altered entrainment. One factor that may modulate and alter the circadian clock in TAMs is tumor fibrosis, which is known to occur particularly in pancreatic cancer. ^116^ Factors associated with fibrosis, including matrix stiffness and TGFβ, were shown to modulate the molecular clock in a cell-type specific fashion. Additional investigation into how this may alter clocks of cells in tumors and in pre-tumor chronically inflamed tissue, particularly how it affects immune cells, could be fruitful.

Genetic ablation of the macrophage circadian clock dysregulates macrophage metabolism and promotes tumor growth, ^117^ underscoring the potential importance of TAM circadian rhythms in regulating their role in the tumor microenvironment. However, the role of the circadian clock in regulating TAM phenotype and function remained unclear. Nonetheless, if additional aspects of macrophage phenotype are shown to vary by time of day in TAMs, this could inform on using chronotherapy (treatment timed in a circadian fashion to maximize efficacy) that is aimed at reprogramming TAMs. Further studies on circadian regulation of macrophage functions within the tumor microenvironment relative to peripheral tissues will arm us with the information we need to employ chronotherapeutic-based approaches of immunotherapy treatments for cancer.

## Application of macrophage circadian biology to the clinic

Due to the major role of TAMs in promoting immune suppression, some approaches to cancer therapy have focused on re-polarizing macrophages from a pro-tumorigenic, pro-resolution state to an anti-tumorigenic, pro-inflammatory state to alleviate macrophage-mediated immune suppression in the TME and further promote anti-tumor immunity. ^118^ There are numerous TLR agonists currently in clinical trials for combination with immune checkpoint therapy, as well as inhibitors of CD47/SIRPa to block efferocytosis of cancer cells by macrophages. ^119^ Given observations of time-of-day dependency in macrophage response to stimuli, leveraging time-of-day variations in targets could be promising avenue to increase efficacy, as showcased by recent studies using PD-1/PD-L1 ICB. ^1^^,^^2^

**Figure 3.**
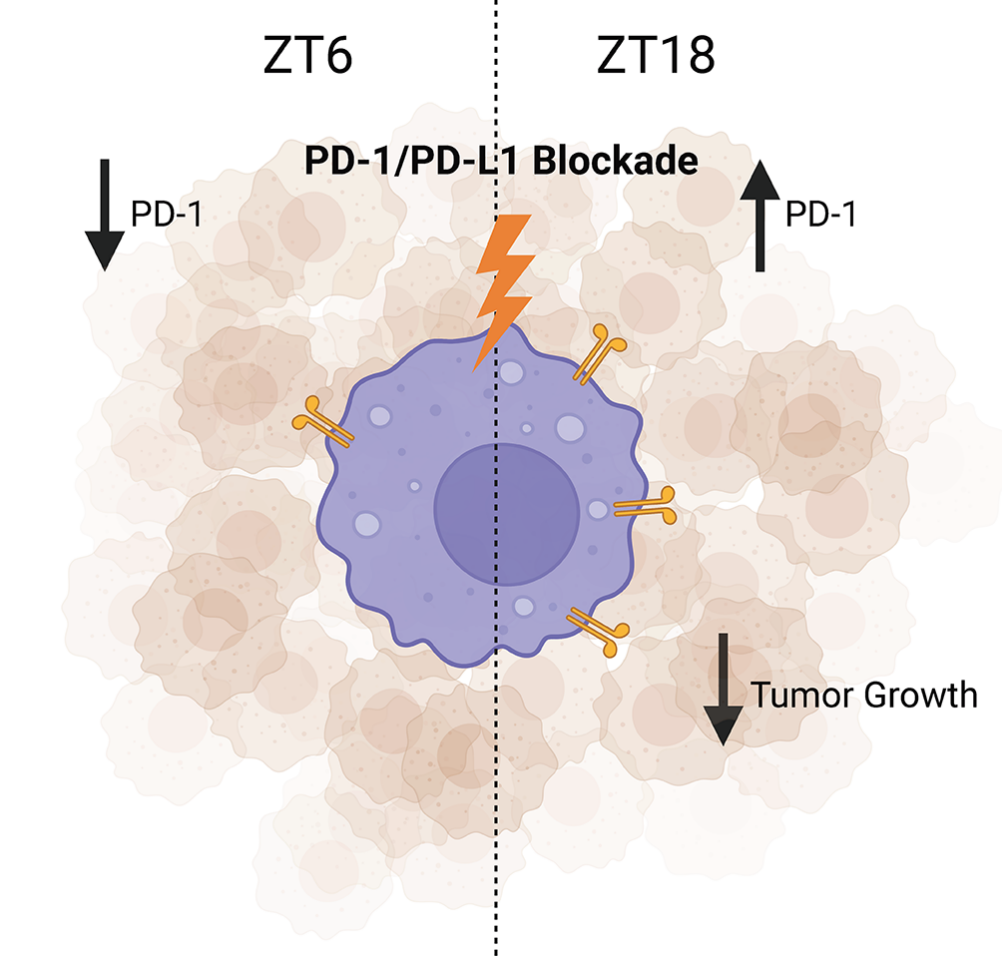
Circadian expression of the checkpoint protein PD-1 by macrophages within the tumor and PD-1/PDL-1 blockade therapy efficacy. In B16/BL6 (melanoma) tumor-bearing mice, the administration of PD-1/PD-L1 inhibitor during the dark phase (ZT16) results in higher anti-tumor effects when compared with the same treatment administered during the light phase (ZT8). Figure created with BioRender.com. PD-1, programmed cell death protein 1; PD-L1, programmed death-ligand 1.

In humans and mice, frequency of PD-1+TAMs increases with disease stage and tumor progression, respectively. ^120^ Expression of PD-1 by TAMs has been shown to promote tumor progression by facilitating induction of oxidative metabolism that supports pro-tumorigenic and anti-inflammatory functions. ^86^ As such, PD-1+ TAMs expressed high levels of CD206, ARG1 and IL-10, relative to their PD-1 counterparts. By contrast, blocking engagement of macrophage PD-1 by PD-1/PD-L1 blockade or deletion of macrophage PD-1 resulted in a shift toward glycolytic metabolism and elevated expression of cytokines associated with a pro-inflammatory macrophage phenotype. This suggested that the skewing of TAM population phenotype from pro-tumorigenic to anti-tumorigenic following anti-PD-1/PD-L1 treatment in various tumor models is due to direct effects on macrophages in addition to indirect effects from T cell-dependent alterations. ^86^^,^^121^ These findings are particularly intriguing, as circadian patterns in frequency of PD-1+ TAMs has been observed in B16/BL6 (melanoma) tumor-bearing mice ( Figure 3). ^1^ Taking a chronotherapeutic approach, increased efficacy of PD-1/PD-L1 ICB therapy was observed when administered at the time of day when PD-1+ TAMs were most frequent (in the middle of the active phase) compared to when treatment was administered at time of day when PD-1+ TAMs were lowest (in the middle of the inactive phase). ^1^ These data suggest that the phenotype of TAMs can vary by time-of-day, and that synchronizing treatment to take advantage of these rhythms could be beneficial.

Of note, these data were confirmed in a recent retrospective study of human melanoma patients who received ICB therapy. Those patients who received less than 20% of their ICB infusions before 4:30 PM (near the end of the human active phase) had a statistically significant increase in overall survival as compared to those who received more than 20% of their infusions after 4:30 PM, ^2^ mirroring findings from mice, though circadian trafficking of T cell populations likely also played a major role in this observation. Further investigation of whether frequency of PD-1+ TAMs is circadian across different types of cancer, and whether this is true in humans as well as mice, will help further our understanding of the potential application of this therapeutic approach.

Separately, other groups have experimented with delivering IL-12 intratumorally to repolarize macrophages and other immunosuppressive myeloid cells towards a pro-inflammatory phenotype. This improved the efficacy of radiotherapy, eliciting a more potent anti-tumor immune response. ^122^ Knowledge of how circadian rhythms control macrophage phenotype and function, especially in the TME, may inform clinicians on the most effective use of these approaches.

## Conclusions, open questions, and future perspectives

Overall, current evidence, however limited, suggests that exploiting circadian regulation of key macrophage-derived immunoregulatory factors may allow us to seek further benefits from clinical treatments targeted to these factors. However, we know very little about how circadian regulation of macrophages is affected by cancer.

One challenge that remains is determining if macrophages within tumors have normal or disrupted circadian rhythms. Computational analysis has suggested that molecular clocks are disrupted in human tumor samples across 12 different types of cancer. ^123^^,^^124^ As this analysis was performed using transcriptomic data from whole tumors, factors that cause disruption of the molecular clock in the TME, and cells in which the clock is disrupted, remains unclear. Several metabolically stressful factors associated with the TME such as nutrient limitation, hypoxia, and acidic pH can disrupt the molecular clock. ^125^^–^^128^ This is likely to change across tumor progression, since when the tumor is still small it will be relatively well-vascularized and thus have less hypoxia, less acidic pH and maybe less nutrient limitation. However, as the tumor progresses and grows larger, it would conceivably become less well-vascularized and will thus begin to have areas of hypoxia and acidic pH and possibly also nutrient limitation. How this affects circadian regulation of immune cells within the TME is unknown. There is ample evidence that these same stressors can influence immune cell phenotype and function, but the role of the clock in these phenotypical and functional changes is unknown. ^97^^,^^129^^–^^132^ As this may influence how clinicians plan to deliver TAM-modulating therapies in a chronotherapeutic fashion, this is a possible subject of future study.

In conclusion, an emerging body of literature suggest that macrophage phenotype and function are heavily influenced by circadian rhythm control. The molecular clock influences and tunes functions such as cytokine secretion, efferocytosis, phagocytosis, surface marker expression, and interaction with other immune cell types such as T cells. This molecular clock control may be influenced in part by internal metabolic shifts and changes in external metabolic cues. TAMs are heavily skewed towards a pro-resolution phenotype that leads to suppression of the anti-tumor immune response and direct pro-tumorigenic activity, and this is shaped by changes in the TME. A better understanding of how macrophage circadian rhythms are modulated by the TME will aid in determining how this pathway contributes to the pro-tumorigenic nature of macrophages. More importantly, knowledge of TAM circadian rhythms may aid in therapy decisions that directly and indirectly target macrophages, by using time of day information to maximize efficacy.

## Data Availability

No data are associated with this article.
