# Supplementary material for: Do macrophages follow the beat of circadian rhythm in TIME (Tumor Immune Microenvironment)?
Source: F1000Res. 2023 Jan 27;12:101. [Version 1] doi: 10.12688/f1000research.129863.1 (PMC10352629; doi:10.12688/f1000research.129863.1)
Supplement: Peer review history

# Peer review: Reviewer response for version 1

**DOI:** 10.5256/f1000research.142579.r181754

**Published:** 2023-07-17

**Authors:** Kristin Eckel-Mahan

**Affiliations:** 1 Brown Institute of Molecular Medicine, McGovern Medical School at the University of Texas Health Sciences Center at Houston, Houston, TX, USA

In “Do macrophages follow the beat of circadian rhythms in TIME (Tumor Immune Microenvironment)?” Knudsen-Clark *et al. *provide a nice review of the role of circadian rhythms in the tumor microenvironment in tumor progression. Specifically, the authors do a nice job of highlighting circadian regulation of immune response at the level of macrophage activity. The article highlights the heterogeneity of tumor- and tumor microenvironment-associated macrophages at the level of activity and circadian function. I think the authors could discuss the molecular clock of macrophages in more detail, and highlight some of the initial findings underscoring a role of the circadian clock within this cell type.

 Comments:

1. In the first section, where authors describe the dysregulation of immune response in cancer, the author may wish to further explain what the “pro-resolution” state means in the context of macrophage function. Some of the material from the “contextual cues dictate macrophage function” might be introduced earlier as it relates to the resolution phase. Furthermore, some additional details related to M2 vs. M1 states would be helpful for those outside of the field.
2. Related to point #1, some of the background in the next section related to macrophages might be placed earlier in the review.
3. In discussing some studies, the authors should provide more experimental and molecular details. In places, the discussion is rather vague.
4. I get the impression that the authors wish to avoid describing the molecular clock. However, I think in doing so, they could add more details related to disruption of immune response in macrophages by the presence or absence of specific clock proteins (CRYs vs. BMAL1, for example).
5. The authors could expand the last section discussing the potential for clinical relevance, and discuss the half life and properties of currently used PD-L1 inhibitors (for example) since they focus on the potential for time-of-day response.

Minor Concerns:

1. A period appears to be missing in the abstract before the sentence starting with, “circadian rhythms”.
2. The authors can probably eliminate the reference to ZT0 as “usually 7 AM”, as this will vary from facility to facility. Describing ZT0 as “lights on” or “beginning of the resting/light phase” should be sufficient.
3. Missing word in the sentence after reference 70.

Is the review written in accessible language?

Yes

Are all factual statements correct and adequately supported by citations?

Yes

Are the conclusions drawn appropriate in the context of the current research literature?

Yes

Is the topic of the review discussed comprehensively in the context of the current literature?

Yes

Reviewer Expertise:

circadian rhythms

I confirm that I have read this submission and believe that I have an appropriate level of expertise to confirm that it is of an acceptable scientific standard.

# Peer review: Reviewer response for version 1

**DOI:** 10.5256/f1000research.142579.r161785

**Published:** 2023-02-20

**Authors:** Carla Finkielstein

**Affiliations:** 1 Integrated Cellular Responses Laboratory, Department of Biological Sciences and Center for Drug Discovery, Fralin Biomedical Research Institute, Virginia Polytechnic Institute and State University, Roanoke, VA, USA

In this review article, the authors effectively summarize a large volume of information regarding the many roles that macrophages play as components of the tumor immune microenvironment (TME) and during tumor growth. Furthermore, the authors incorporate findings that relate to the circadian biology of macrophages and discuss their relevance in the context of metabolism, immune remodelling, and tumor growth. More importantly, they stress the importance of more research in macrophages’ clock to better exploit their different functionalities therapeutically.

 This reviewer believes the article is well-written, contains updated and critical information, and will be highly cited based on its quality.

 A number of minor recommendations are listed below:

1. Introduction: Remove the sentence “We will investigate……well-characterizes.” The sentence is redundant with the info provided in the same paragraph.
2. The section in “Dysregulation of the immune response…” would benefit from a comprehensive figure that summarizes the information in that section.
3. In the same section, the authors introduce the “immune checkpoint blockade therapy”; however, there is not any explanation what this is all about. This reviewer believes the reader would benefit from a few sentences that explains what ICB therapy is all about.
4. To me, it seems that there is a figure missing between Fig. 1 and Fig. 2 that summarizes the many functions of macrophages and how they are regulated. The information is overwhelming, and the reader would benefit from a summary figure.
5. There are instances in which the data discussed in the article merits the addition of specific information such as whether the data were collected from mice or humans specimens. For example, when discussing the chemokine and cytokine secretion by macrophages in response to stimuli and at specific phases of the day.
6. Figure 2 is referenced within the first sentence of the “Macrophages in tumorigenesis” section. However, the section is incredibly richer in information that is not depicted in Figure 2. The way that figures are embedded in the manuscript look like the authors needed to add figures to the review but they do not take good advantage of those figures to convey complex information to the reader. I suggest to revisit the info in figures and integrate the text and figures better.
7. A "." is missing in the abstract between the words "response Circadian rhythms".

Is the review written in accessible language?

Partly

Are all factual statements correct and adequately supported by citations?

Yes

Are the conclusions drawn appropriate in the context of the current research literature?

Yes

Is the topic of the review discussed comprehensively in the context of the current literature?

Yes

Reviewer Expertise:

circadian rhythms, cancer, cell cycle, DNA damage, chronotherapy

I confirm that I have read this submission and believe that I have an appropriate level of expertise to confirm that it is of an acceptable scientific standard.
